# Co-Producing Phycocyanin and Bioplastic in *Arthrospira platensis* Using Carbon-Rich Wastewater

**DOI:** 10.3390/biotech12030049

**Published:** 2023-07-03

**Authors:** Hajar Shayesteh, Damian W. Laird, Leonie J. Hughes, Mohammad A. Nematollahi, Amin Mirshamsi Kakhki, Navid R. Moheimani

**Affiliations:** 1 Centre for Water, Energy and Waste, Harry Butler Institute, Murdoch University, Murdoch 6150, Australia or hajar.shayesteh@mail.um.ac.ir (H.S.); n.moheimani@murdoch.edu.au (N.R.M.); 2 Department of Biotechnology and Plant Breeding, Faculty of Agriculture, Ferdowsi University of Mashhad, Mashhad 91779-48978, Iran; mirshamsi@um.ac.ir; 3 School of Mathematics, Statistics, Chemistry and Physics, College of Science, Technology, Engineering, and Mathematics, Murdoch University, Murdoch 6150, Australia; 4 Department of Fisheries, Faculty of Natural Resources, University of Tehran, Karaj Campus, Tehran 77871-31587, Iran; malahi@ut.ac.ir

**Keywords:** *Arthrospira platensis*, bioplastic, bioremediation, phycocyanin, waste organic carbon

## Abstract

Microalgae can treat waste streams containing elevated levels of organic carbon and nitrogen. This process can be economically attractive if high value products are created simultaneously from the relatively low-cost waste stream. Co-production of two high value microalgal products, phycocyanin and polyhydroxybutyrate (PHB), was investigated using non-axenic *Arthrospira platensis* MUR126 and supplemental organic carbon (acetate, oxalate, glycerol and combinations). All supplemented cultures had higher biomass yield (g/L) than photoautotrophic control. All cultures produced PHB (3.6–7.8% *w*/*w*), except the control and those fed oxalate. Supplemented cultures showed a two to three-fold increase in phycocyanin content over the eight-day cultivation. Results indicate co-production of phycocyanin and PHB is possible in *A. platensis*, using mixed-waste organic carbon. However, supplementation resulted in growth of extremophile bacteria, particularly in cultures fed glycerol, and this had a negative impact on culture health. Refinement of the carbon dosing rate is required to minimise impacts of native bacterial contamination.

## 1. Introduction

There is an increasing market to develop sustainable technologies for production of safe and environmentally friendly products such as bioplastics and pigments. However, these products also need to be produced in a way that is both financially feasible and environmentally sustainable [1]. Microalgae, including cyanobacteria, have been shown to produce a wide variety of compounds with biotechnological applications in areas such as agriculture (food, animal feed, biofertilizer), bioremediation, bioenergy, and renewable fuel production and human health [2,3]. In fact, several high-value chemicals from microalgae and cyanobacteria are already commercially exploited, such as β-carotene, astaxanthin, phycocyanin, and polyunsaturated fatty acids (PUFA) [4]. Among the many species of cyanobacteria, *Arthrospira platensis* has attracted considerable interest from the health food industry because of its valuable components such as proteins and vitamins and, more importantly, C-phycocyanin (C-PC) [5,6,7]. This photosynthetic pigment is a nitrogen-storing protein-pigment complex and has been shown to have anticancer [8], antioxidant [9], and neuroprotective [10] properties. The unique blue colour and fluorescent properties of phycocyanin make it a novel natural colorant that can be used in food and cosmetic applications [11], and as a medical imaging agent [12].

In addition to their exploitation for pigment production, cyanobacteria are gaining attention as a source for low-cost biopolymers [13]. Polyhydroxyalkanoates (PHAs) have similar thermal and mechanical properties to petroleum-derived bioplastics but are biodegradable and biocompatible. Polyhydroxybutyrate (PHB) is a type of homopolymeric PHA where the monomer is 3-hydroxybutyrate (3HB) [14]. The mechanism of PHA synthesis in microalgae involves the conversion of acetyl-CoA into the monomer 3-hydroxybutyrl-CoA by the enzyme acetoacetyl-CoA reductase (PhaB). This reaction is followed by the polymerisation of 3-hydroxybutyryl-CoA monomers by the enzyme PHB synthase (PhaC), leading to the formation of PHB polymer [15]. PHB granules are then stored in the cytoplasm of the microalgae as a carbon storage product [16]. PHB has a wide range of potential applications in biomedicine (scaffold for tissue engineering), pharmacology (encapsulation of therapeutics), agriculture (encapsulation of fertilisers) and biodegradable consumer packaging [17].

Heterotrophic bacteria have long been known to produce PHA from excess carbon when growth is restricted due to limited availability of other nutrients [18]. As photosynthetic prokaryotes, cyanobacteria can also synthesise PHA, have a short life cycle, have simple nutrient requirements for their growth, and can be cultured in a heterotrophic/mixotrophic fashion [19]. However, production of PHB in most cyanobacteria is accomplished using CO_2_ as the only carbon source and both yield and productivity is generally low. A notable exception is *Synechococcus* sp. MA19, which accumulates up to 55% (*w*/*w*) PHB of the dry cell mass under phosphate-limited conditions at 50 °C [20]. Supplementation of cynanobacterial cultures with short chain carbon sources, e.g., acetate, can lead to an increase in PHB accumulation when compared to growth under strictly photoautotrophic conditions. For example, yield increases of up to 35% (*w*/*w*) in *Nostoc muscorum* [21] and 10% in *A. platensis* UMACC 161 [22] have been reported.

Photoautotrophic culture of *A. platensis* in open raceway ponds is the conventional method for production of C-PC using *A. platensis* [23]. However, these cultures usually have low biomass productivity, which increases the cost of harvesting cells and eventual product extraction. The mixotrophic culture of cyanobacteria using waste carbon sources is one possible alternative for increasing cell concentration and, more importantly, reducing the overall cost of microalgal biomass production [24,25]. This would be particularly attractive if the carbon source was a waste product from another process. For example, glycerol, as a by-product of the biodiesel production process, is considered a low cost and renewable feedstock [26]. Similarly, oxalate, a major by-product of alumina production, is toxic in high concentrations and requires treatment before release to the environment which, in turn, involves additional costs [27]. As *A. platensis* is known to grow utilising non-CO_2_ carbon sources, consumption of organic compounds such as glycerol and oxalate would result in reuse of ‘waste’ carbon, saving money and resulting in microalgal biomass, which could be used for other purposes. Several studies have reported that *A. platensis* cultures are able to grow in the presence of individual organic carbon sources. Successful growth under axenic conditions has been reported for glucose [28,29,30,31,32] and glycerol [33]. Acetate [34] and oxalate [35] have been utilised in *A. platensis* cultivation under non-axenic conditions.

The production of multiple bio-products from a single organism inevitably means that maximum productivity of one of the products is reduced. However, if both of those products are of high value and can be produced from low-cost inputs, a drop in the yield of one product may be justified. If a decrease in product yield is possible then it is important that the organism being utilised can be cultured at a scale that minimises the impact of that decrease. *A. platensis* has a long history of commercial production at a large scale, where conditions are optimised for biomass production. Considering the cultivation of *A. platensis* in a biorefinery context, it may be possible to generate additional revenue streams if conditions that optimise production of multiple high-value products are available. Concurrent production of both PHB and C-PC has been reported from *Nostoc muscorum* in mixotrophic culture supplemented with 0.4% fructose and glucose [36]. In a previous study, we have shown accumulation of polyhydroxybutyrate (up to 3% dry weight) and phycocyanin (up to 145 mg/g) in *Arthrospira platensis* MUR126 under mixotrophic cultivation and nutrient (N and P) starvation [35]. In that work it was suggested that increased co-production of these bio-compounds could be achieved by providing a minimum level of N, as well as a greater concentration of organic carbon.

In this study, our aim was to investigate the generation of multiple bio-products from *A. spirulina* using a combination of common agro-industrial waste organic carbon compounds (acetate, oxalate, glycerol and their mixtures) and pulse-feeding of those carbon sources. Success was measured by determining the effects on biomass, C-PC and PHB accumulation in *A. platensis*.

## 2. Materials and Methods

### 2.1. Microalgae Species, Inoculum Preparation and Culture Condition

*Arthrospira platensis* MUR 126 from a non-axenic outdoor pond culture located at the Murdoch University Algae R&D Centre was used for experiments.

All indoor experimental cultures were supplemented with organic carbon under fed- batch cultivation at a temperature of 25 ± 2 °C, illuminated at ~300 μmol m^−2^ s^−1^ on a 12:12 light-dark cycle. Light was switched on at 06:00 and off at 18:00. Oxygen was provided by continuous bubbling with atmospheric, unfiltered ambient air at 2 L/min. Experiments were conducted by inoculating autoclaved Zarrouk medium [37] with ‘centrifuged’ biomass from the non-axenic *A. platensis* culture. 50 mL samples from the outdoor culture were centrifuged at 3000 rpm for 10 min (Juan C142 benchtop centrifuge), washed with deionised water and the biomass added to 300 mL of autoclaved Zarrouk medium contained in a 500 mL Schott™ flask, to produce an initial yield of 1.45 ± 0.10 gL^−1^. The relatively high biomass concentration was to minimise potential biological contamination as described by Ogbonna et al. [38]. No acclimatisation of the biomass to indoor, low light conditions, etc. occurred prior to inoculation.

Each treatment consisted of 3 replicates and 40 mL samples for analysis were taken at 0, 2, 4, 6, and 8 days after inoculation. No additional Zarrouk media was added to the flask after samples were removed.

Treatment cultures were provided with 15 mL of supplementary carbon source (acetate, oxalate, glycerol, glycerol + oxalate, glycerol + oxalate + acetate), representing a total carbon addition of 20 mM. No additional nutrient/Zarrouk media was added at this time. The choice of C concentration was determined based on known toxicity of acetate to *A. platensis* under mixotrophic cultivation [35].

The carbon source was added twice daily at 06:00 and 18:00 to correspond with the start and end of illumination. This was done to address the possibility that PHB may be catabolised during the dark cycle [35] when conditions could become carbon-limited. Concentrations of the supplementary carbon sources added were acetate (10.0 mM), oxalate (10.0 mM), glycerol (6.67 mM), mixture of glycerol (3.34 mM) and oxalate (5.00 mM), and the mixture of glycerol (2.22 mM), oxalate (3.33 mM) and acetate (3.33 mM). The acetate concentration tested was based on a level known to be suitable for *A. platensis* cultivation [35]. The concentration of the other single and mixed carbon sources was scaled to ensure equivalent moles of C with the acetate-only supplementation, e.g., acetate and oxalate both contain two carbons so the tested concentration of both was the same. Cultures provided with phototrophic-only conditions, i.e., no additional nutrient source, were used as a control.

### 2.2. Culture Productivity and Health

Dry weight (DW) and ash-free dry weight (AFDW) were measured based on methods previously described in Moheimani et al. [39]. Glass microfiber filters (Whatman™ GF/C 25 mm, cat. number 1822-025) were washed with deionised water, dried at 90 °C for 24 h, and weighed. After filtration of 5 mL of harvested culture, filter papers were again dried at 90 °C for 4 h and re-weighed. Filters with dry biomass were then ashed at 450 °C (furnace) overnight and weighed again to determine AFDW. Biomass productivity was calculated as described by Moheimani et al. [39].

Chlorophyll *a* content was determined according to the methods of Jeffrey and Humphrey [40]. A 5 mL sample of the culture was concentrated by filtration with a Whatman™ GF/C filter (25 mm, cat. Number 1822-025). Cell walls were broken by adding liquid nitrogen and chlorophyll *a* extracted using 90% chilled aqueous acetone. Samples were then centrifuged, and the absorbance of the supernatant was measured at 664 and 647 nm (BioMate 3S UV-visible spectrometer) and the amount of chlorophyll *a* was calculated utilising Equation (1):

Chlorophyll *a* (mg/L) = 11.93A_664_ − 1.93A_647_ (1)

### 2.3. Chemical Analyses

#### 2.3.1. Phycocyanin

A 5 mL aliquot of microalgae culture was harvested and filtered with a GF/C filter (Whatman™, 25 mm, cat. number 1822-025). The biomass was subjected to two freeze-thaw cycles, crushed in liquid nitrogen, re-suspended in 5 mL of 100 mM phosphate buffer (pH 7) and refrigerated overnight in the dark. Cell debris was removed by centrifugation (4000 rpm, 10 min) and the absorbance of the supernatant was measured at 615 and 652 nm (BioMate 3S UV-visible spectrophotometer). C-PC concentration was calculated using Equation (2) established by Bennett and Bogorad [41]:

C-PC (g/L) = (A_615_ − 0.474 × A_652_)/5.34 (2)

#### 2.3.2. Polyhydroxybutyrate (PHB)

A 10 mL aliquot of culture was centrifuged and the biomass pellet was stored in a freezer (−20 °C) before freeze-drying overnight. PHB was extracted and analysed as the butyl ester by GC-MS as detailed in Nematollahi et al. [35], using benzoic acid as an internal standard.

### 2.4. Statistical Analysis

Statistical analysis of data was performed using SigmaPlot software (version 13). One-way analysis of variance (ANOVA) was used to determine significant differences between treatments and a 5% probability level was considered significant with a sample size of 3. All measurements were expressed as means ± standard error (SE).

## 3. Results and Discussion

### 3.1. Biomass and Pigment Production

**Figure 1:**
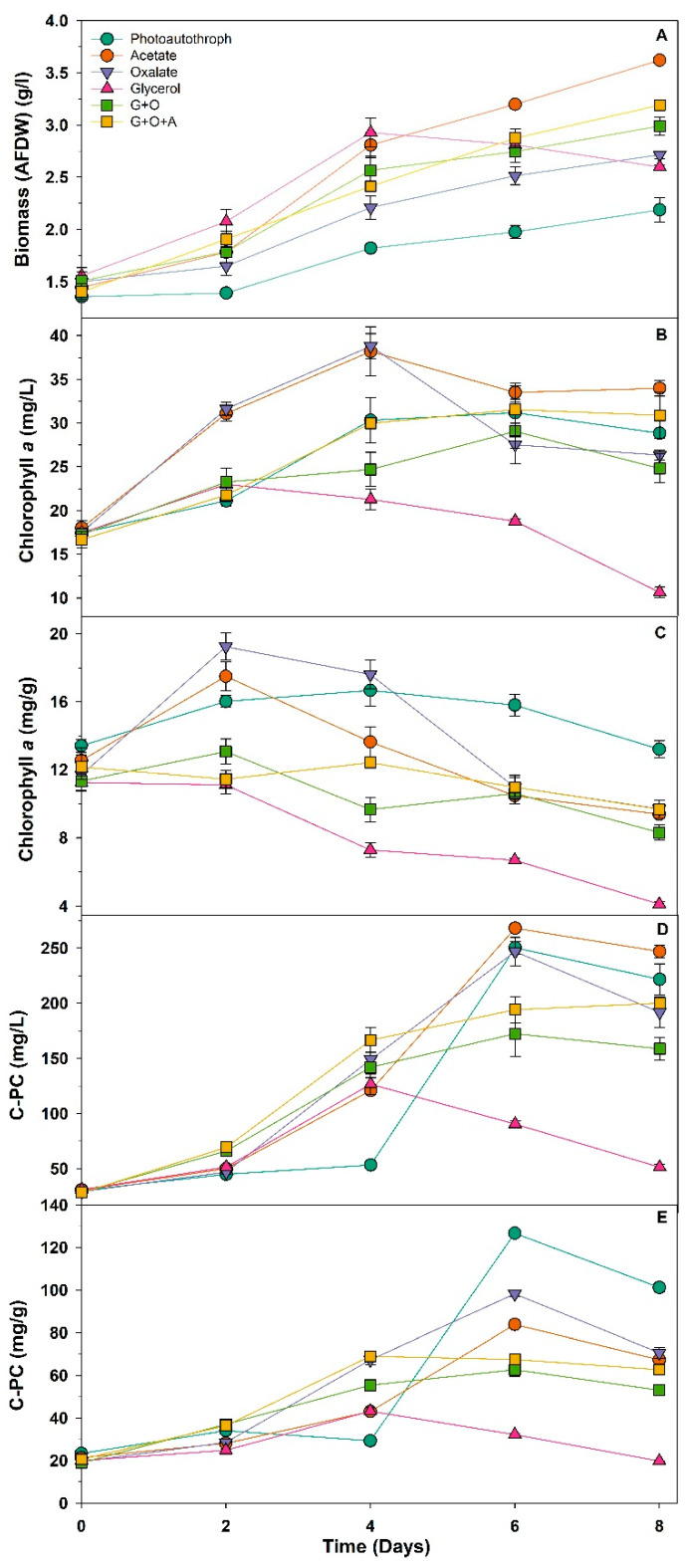
Biomass (**A**), chlorophyll *a* (**B**,**C**) and phycocyanin (**D**,**E**) concentration and content of *A. platensis* cultures fed with different organic carbon sources; photoautotroph (green circle), acetate (red circle), oxalate (purple triangle), glycerol (pink triangle), glycerol + oxalate (green square), and glycerol + oxalate + acetate (yellow square). Data mean ± SE, *n* = 3.

All cultures, including the photoautotrophic control, showed an increase in biomass (AFDW) between day 0 and day 4 (Figure 1A). At day 4, all the carbon-supplemented cultures had a statistically significant increase in biomass compared to the control (One-way ANOVA, *p* < 0.05). The largest change in biomass was observed in the glycerol and acetate-fed cultures. However, there was a noticeable change in biomass for the glycerol culture from day 4–8 of cultivation (Figure 1A). The day 8 biomass concentration for *Arthrospira* grown on the glycerol-only culture was 11% lower than the day 4 maximum (Figure 1A). The control photoautotrophic culture and all cultures supplemented with small organic acids (e.g., acetate and oxalate) continued to exhibit increased biomass growth over days 4–8. However, except for the acetate-only culture, the biomass yields at day 8 for all other treatments are equal to, or lower than that for the glycerol-only culture at day 4 (Figure 1A). The specific growth rate (μ), μ_max_, and maximum biomass productivity for the treatments trended in a similar manner: always highest and lowest in glycerol-only and photoautotrophic cultures, respectively (Table 1).

Volumetric chlorophyll content (mg per L of culture) in the glycerol-only culture increased between day 0 and day 2 but then trended downward to day 6 before halving between day 6 and 8 (Figure 1B). Overall, the chlorophyll content in the glycerol-supplemented culture was less than half of that measured on day 0. The *Arthrospira* control culture (photoautotrophic growth) and those supplemented with small organic acids all had an overall increase in chlorophyll content by day 8 of cultivation (Figure 1B). However, this was not strictly linear, nor consistent, between organic acids. Oxalate-only and acetate-only cultures reached a maximum volumetric chlorophyll concentration at day 4 and then fell by 29 and 12%, respectively, by day 6 and there was no significant change in volumetric chlorophyll measurements at day 8 (Figure 1B). The photoautotrophic control glycerol + oxalate (G + O), and the glycerol + oxalate + acetate (G + O + A) supplemented cultures reached maximum chlorophyll concentrations at day 6. Volumetric chlorophyll concentrations then decreased slightly between days 6–8 for the photoautotrophic and G + O cultures (Figure 1B).

Changes in chlorophyll content on a mass basis (Figure 1C) were, perhaps, more instructive of the changes occurring in each of the carbon-supplemented cultures. The mass-based chlorophyll content in the control photoautotrophic culture was essentially unchanged over the period of cultivation: there was a slight increase to day 4 but this was not statistically significant, and by day 8 levels were back to those measured on day 0. In contrast, the chlorophyll content of the supplemented cultures showed much larger changes with the measured chlorophyll content for all statistically significantly lower at day 8 than day 2 (*p* < 0.001). Cultures supplemented with acetate-only (A), oxalate-only (O), and glycerol/oxalate (G + O) all reached maximum chlorophyll content on day 2 before falling steadily to day 8 (Figure 1C). However, the G + O + A culture reached maximum chlorophyll content at day 4 before a gradual decline to day 8 (Figure 1C). The glycerol culture showed a statistically significant continual decline in chlorophyll content from day 0 through day 8 (*t* test, *p* < 0.05) to a point where the day 8 value was only ≈36% of that at the beginning of the cultivation.

Except for the glycerol-only culture, the volumetric yield (mg per L of culture) of phycocyanin was not statistically different between carbon-supplemented and control cultures (Figure 1D) at the end of cultivation. There was an overall 5–8-fold increase for the control and all treatment cultures, except that supplemented with glycerol-only. All carbon-supplemented *Arthrospira* cultures, except glycerol (G), had increased phycocyanin yield from day 2, with maxima achieved at day 8 for G + O + A and day 6 for acetate-only, oxalate-only, and G + O fed cultures (Figure 1D). Maximum phycocyanin yield of *Arthrospira* in the glycerol-only culture was reached at day 4 before falling back to day 0 values by the end of the cultivation period. *Arthrospira* phycocyanin yield in the photoautotrophic culture showed a lag compared to the carbon-supplemented cultures—measured values were constant up to day 4, before a large increase to a maximum at day 6, followed by a slight reduction by day 8 (Figure 1D).

A similar trend was observed when *Arthrospira* phycocyanin content was recorded on a per mass basis (Figure 1E). None of the carbon-supplemented *Arthrospira* cultures achieved the same phycocyanin content as the *Arthrospira* control culture (photoautotroph) at day 8. However, there was still an increase of between 2–3.5-fold in the phycocyanin content from day 0 values for all carbon-supplemented cultures, except those fed glycerol. Results from the glycerol-only culture were again starkly different from all others, with a modest maximum detected at day 4 before levels decreased by day 8 to those recorded at the beginning of cultivation. The culture began to lose its typical vibrant green colour and became much more yellow (see figure in Supplementary Information), suggesting a fault in pigment production and a culture under stress. A drop in *Arthrospira* biomass concentration for the glycerol culture over days 4, 6 and 8 confirmed that a change to the culture was underway (Figure 1B). The observed colour change in the glycerol-only-supplemented *Arthrospira* culture appears to have been due to a severe reduction in the amount of pigment produced (Figure 1B–E), reflected in both phycocyanin yield (Figure 1D) and content (Figure 1E).

It is well known that mixotrophic conditions boost growth and metabolite production in a number of axenically cultivated microalgal genera, including *Arthrospira* [32,42,43,44,45,46,47,48], *Chlorella* [49], *Nannochloropsis* [50] and *Chaetoceros* [51]. Hence, it was not surprising that the *A. platensis* biomass increased under most of the mixotrophic culture conditions, and that biomass yield was significantly higher than that achieved under photoautotrophic conditions. However, the reductions in biomass, chlorophyll and phycocyanin in the glycerol-only culture suggested that something may be affecting culture health and/or metabolism. Additionally, all the other mixotrophic conditions were producing increased biomass growth compared to photoautotrophic growth conditions but there was no correlating decrease in chlorophyll and phycocyanin for those cultures. These results could be explained by: (a) nitrogen chlorosis, (b) some biological or chemical contaminant, (c) a change in metabolism from mixotrophic to heterotrophic metabolism, or (d) a combination of these effects.

Nitrogen chlorosis, a phenomenon where cells gradually change their colour from blue-green to brownish yellow due to nitrogen starvation, has been described in cyanobacteria, particularly in *Synechocystis* sp. cultures [52], and it has been suggested by Duangsri et al. [53] that this yellowing could be due to phycobiliprotein degradation in *A. platensis*. However, data collected here does not allow us to confirm if nitrogen depletion in the culture was responsible for the noticeable colour change.

Biological contaminants, such as bacteria, proliferate in the presence of organic carbon sources even in mixotrophic unialgal cultures [54]. Various bacterial genera have been reported from *A. platensis* cultures in the lab and in ponds [55,56], including halophilic *Halomonas*: a genus able to utilize ‘difficult’ organic substrates such as oxalate and formate. Qualitative culturing of centrifuged algal medium from the mixotrophic cultures on nutrient agar plates revealed the presence of bacteria (Table S1, Figure S2—Supplementary data). The highest number of visible bacterial colonies were in algal cultures supplemented with glycerol alone, and lowest in the cultures supplemented with oxalate, either in combination or alone. In their recently published studies on the cultivation of *A. platensis* in crude and pure glycerol, both Corrêa and Teixeira [54] and Markou et al. [57] reported significant bacterial contamination of the microalgal culture. Bacterial contamination was more pronounced when using pure glycerol compared to crude glycerol. Markou et al. [57] found that bacterial contamination continued to increase with increasing glycerol concentration (0.5–9 g/L). Algal biomass concentration was not greatly affected up to 1.5 g/L glucose but declined rapidly at concentrations greater than 6 g/L glucose. Corrêa and Teixeira [54] also investigated the effect of glycerol concentration on bacterial contamination by holding the initial inoculum biomass constant (0.45 g/L) and varying the amount of supplemental glycerol (0.5–6.14 g/L), finding that increasing glycerol can lead to increased biomass contamination. The initial *A. platensis* biomass concentrations in our study range from 1.33–1.55 g/L (2.2 and 3.5 times higher than Markou et al. [57] and Corrêa and Teixeira [54], respectively) and is consistent with the suggestion from Corrêa and Teixeira [54] that increased initial biomass leads to greater bacterial contamination issues. However, whether utilising the relatively low initial biomass concentration (~0.5 g/L) is feasible for commercial exploitation remains to be ascertained. The glycerol concentrations in our work (2.22–6.67 mM = 0.2–0.6 g/L) are at the lower end of both the Markou et al. [57] and Corrêa and Teixeira [54] studies but were still, apparently, enough to result in a significant reduction in biomass, chlorophyll and phycocyanin production in the culture supplemented with glycerol only.

We chose to use non-axenic cultures in this study to mimic what might occur during large scale, outdoor cultivation of *A. platensis.* When cultivating microalgae at scale in outdoor conditions, some form of bacterial contamination is almost inevitable, and this study suggests that may be exaggerated if cultures are supplemented with readily consumed small organic sugars. The decision to adopt a fed-batch approach was influenced by our recent experience in utilising a single bolus addition approach [35]. In that work, the additional carbon was converted to PHB so quickly that it was then catabolised by the culture by the next sampling date. Additionally, experience with photoautotrophic culture of *A. platensis* suggested that the relatively high pH and alkalinity of the Zarrouk medium would minimise any significant bacterial growth in the non-axenic cultures. Clearly, the fed-batch approach for using these likely waste carbon compounds needs some optimisation/refinement. The bacterial contamination could be mitigated by the use of axenic cultures or the addition of antibacterial agents, but both of these approaches would lead to higher cultivation costs and may not be feasible on the scale required for production of marketable quantities of PHB and phycocyanin.

Other authors have indicated that a fed-batch mixotrophic approach results in much greater biomass productivity for other microalgae, e.g., *Chlorella* and *Nannochloropsis* spp. [45,53]. Control of supplemental carbon delivery is also important in order to avoid supra-optimal carbon levels, with a number of studies showing that a decrease in biomass productivity for mixotrophic cultivations cultivated under such conditions. For example, Chen et al. [34]. reported that an acetate concentration of 4 g/L was optimal for biomass production (as well as a variety of intra-cellular products). At all concentrations below 4 g/L, an increase in fed acetate led to an increase in all measured quantities (e.g., biomass, C-PC, β carotene) but any increase above 4 g/L acetate resulted in a significant decrease in *A. platensis* biomass concentration and compounds of interest. In the present study, the actual concentration of carbon was kept at modest levels and preliminary experiments indicated that all soluble carbon was removed by the culture prior to the next addition (data not shown). The supply of supplementary soluble organic carbon in a fed-batch style enhanced biomass growth and productivity compared to our previous study with a single application of soluble carbon [35].

The response of the mixotrophic cultures (except glycerol-only) are also suggestive of a shift in the equilibrium of mixotrophic and heterotrophic metabolism—no culture-bleaching was evident and the increase in biomass concentration over the 8-day cultivation period was significantly greater than the photoautotrophic control (one-way ANOVA, *p*-value < 0.05). Significant reductions in chlorophyll *a* content as biomass increases are typical of such a metabolic shift. For example, Markou et al. [57] concluded that a significant decrease in chlorophyll concentration recorded at the highest concentrations of added glycerol (6 and 9 g/L) was likely due to a shift toward mixotrophic metabolism, as the alga was no longer solely reliant on the continued synthesis of photosynthetic pigments. Similar results in other microalgal genera have been reported when glucose was supplied to *Chlorella* and *Nannochloropsis* spp. [6,50] and in *Dactylococcus* [58]—increases in dry weight were accompanied by a decrease in chlorophyll *a* due to the reduction of photosynthetic pigment production and a reduction in photosynthetic efficiency.

The results from this study indicate that the fed-batch supplementation of small organic acids and/or sugars leads to *A. platensis* shifting metabolism from purely autotrophic toward mixotrophy or heterotrophy. Using glycerol as a carbon supplement appears to result in a concurrent increase in bacterial load in the culture, and the combination of bacterial growth and shifting algal metabolism can have catastrophic effects on the health of the glycerol-supplemented cultures.

### 3.2. Effects of Organic Carbon Source on Phycocyanin and PHA Production

A healthy culture of *A. platensis* should be actively producing phycocyanin and all the mixotrophic cultures, except that fed glycerol only (G), show an increase in phycocyanin by the end of the culture period (from 40 to 150–250 mg/L). The overall pattern of phycocyanin accumulation generally matches that of the photoautotrophic control, i.e., maximum accumulation at day 6 (150–270 mg/L), followed by some decline from the maxima by day 8. The phycocyanin levels at day 6 trended as A > photoautotroph = O > G + O + A > G + O > G (Figure 1D,E). By the end of cultivation, phycocyanin content in the mixotrophic cultures was significantly lower than that under photoautotrophic conditions on a mass basis (Figure 1E), consistent with the results previously reported in the literature [33,34]. In another study, using a 3-day cultivation, the volumetric yield (mg/L) of phycocyanin remained essentially steady/trended down slightly from photoautotrophic levels (14.2 to 12.8 mg/L) by the end of cultivation when glycerol was added at 0.5–1.5 g/L [57]. However, when glycerol concentrations of 3, 6, and 9 g/L were utilised, phycocyanin levels dropped dramatically, falling to 46% of control levels for supplementation with 3 g/L glycerol and only 9.5% of control levels when 9 g/L of glycerol was utilised [57]. It has also been reported that phycocyanin content in *A. platensis* essentially halved (78.05 to 42.25 mg/g) when pure glycerol concentration was doubled (3.07 to 6.14 g/L) and that reduction was even more pronounced (108.05 to 27.28 mg/g) when crude glycerol was utilised [54]. In the same study it was also found that the maximum phycocyanin levels for all treatments and control, except for glycerol, were closer to the 108.05 mg/g [54] than the 197.2 mg determined by Narayan et al. [33]. Even though the phycocyanin content in our study was less than that for the control at the end of the cultivation period, the phycocyanin yields were at least double, and in some cases triple, those recorded at the beginning of cultivation.

Calculated phycocyanin productivity in this study was not statistically significantly different between the photosynthetic control and those carbon-supplemented cultures containing a majority of acetate or oxalate (Table 1). This suggests that carbon supplementation is not a useful strategy for non-axenic culture of *A. platensis* if focused on maximising phycocyanin yield and productivity. However, Markou et al. [57] have shown that in *A. platensis* cultured using supplementary glycerol the decrease in phycocyanin production is somewhat offset by an increase in the levels of protein and lipids. The combination of a relatively inexpensive carbon source, an increase in protein and lipid content and no substantial effect on phycocyanin productivity may make carbon supplementation an attractive option for large scale *A. platensis* culture based on obtaining multiple products. In fact, Corrêa and Teixeira [54] have suggested that a small reduction in phycocyanin levels may not be an issue if an additional product (in their case a bioplastic) can also be harvested.

Generating high-value products from mixotrophic cultivation of microalgae requires balancing growth of the biomass and production of the desired intracellular products. That often means balancing photosynthetic and respiratory pathways in the cultivated microalgae [58]. In fact, Grama et al. [58] claim that maximum biomass growth may be achieved by finding culture conditions that balance photosynthetic CO_2_ generation and respiratory O_2_ production, i.e., creating a net zero flux of both CO_2_ and O_2_. This is also the case for the production of multiple products from a single microalgal culture, as assimilated carbon can be allocated to growth or pigment production or multiple storage compounds, e.g., polyhydroxyalkanoates (PHAs) or carbohydrates or other lipids. A complex equilibrium exists between all of these interconnected biochemical pathways that are sensitive to numerous biotic and abiotic factors. For example, phycocyanin is a nitrogen-rich storage compound and an accessory pigment, and its production is favoured by relatively high nitrogen supply and low light levels [57]. However, PHA-accumulation is often favoured by N limitation, although it has recently been reported that simultaneous nitrogen depletion and supplementation with glycerol when cultivating *A. platensis* had no effect on levels of bioplastic production [55].

**Figure 2:**
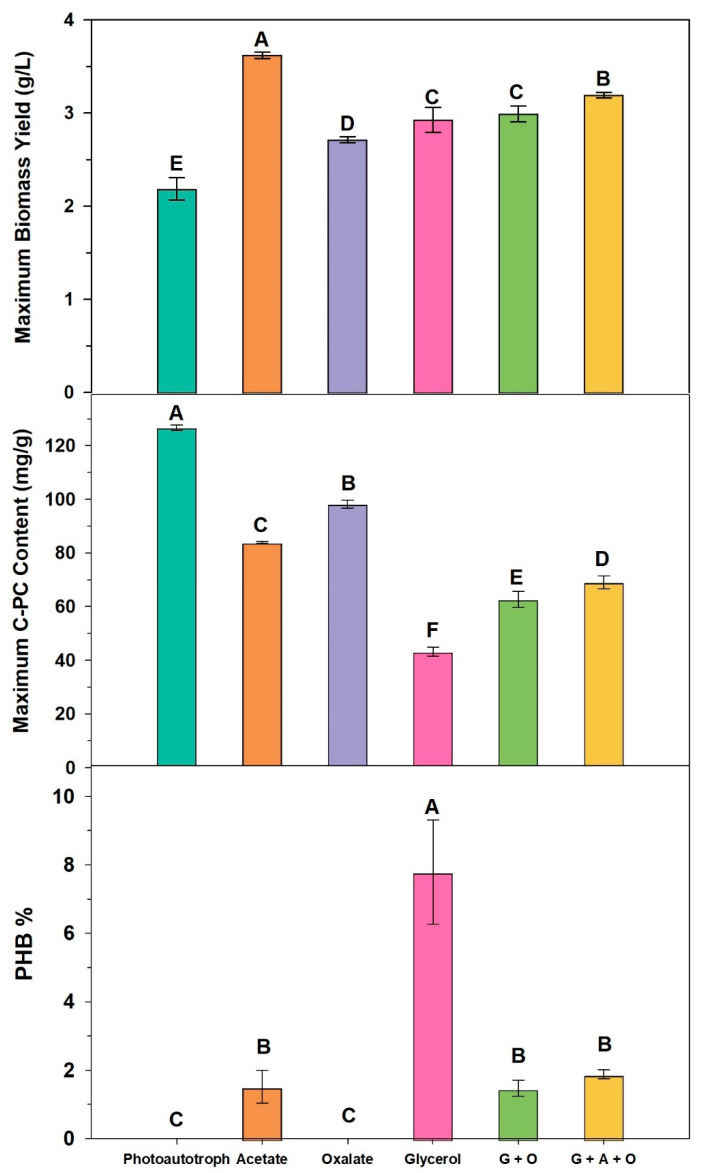
Maximum biomass yield, maximum C-PC content and PHB % of dry weight determined from all cultures for 8 days. Different letters on bars indicate statistically significant differences between treatments (one-way ANOVA, *p* < 0.05, *n* = 3).

PHA in the form of polyhydroxybutyrate (PHB) was detected in quantifiable amounts in all the cultures supplemented with an organic carbon source, except oxalate (Figure 2). The highest PHB content (% by mass) was produced in the glycerol-supplemented culture (one way ANOVA, *p* < 0.05) and is at the higher end of reported PHA levels obtained from *A. platensis* cultures (Table 2). PHB accumulation in *A. platensis* grown under mixotrophic conditions has been reported to vary between 0.1% (1.0 mg/g) and 19.2% (192 mg/g) of cell dry weight (Table 2). However, values up to 350 mg/g are known from other species such as *Synechocystis* (Table 2). The PHB content from the other mixotrophic cultures (i.e., acetate = G + O = G + O + A) were lower than that from the glycerol culture and not significantly different from each other. No PHB was detected in the photoautotrophic control culture, revealing that the additional organic carbon was involved in PHA accumulation. As this compound was measured at the end of the culture period (not tracked on a time course basis), it is not possible to definitively correlate PHB accumulation with the biomass yield at this time.

Comparison of the phycocyanin and PHB yields (Figure 2) suggests that there is a reduction in phycocyanin when PHB is accumulated, although interpretation of this data is complicated by the bacterial contamination observed in all carbon-supplemented cultures.

The largest decrease in phycocyanin (compared to photoautotrophic control) was observed for the glycerol-fed culture, and this culture had the largest PHB accumulation but was also the culture most heavily contaminated with bacteria, leading to actual culture collapse. It could be argued that carbon is being diverted to PHB synthesis and away from phycocyanin accumulation but, unfortunately, we cannot argue that conclusively in this instance. The statistically significant differences in biomass and phycocyanin in acetate, G + O, and G + O + A cultures did not result in similar differences in PHB accumulation. Nevertheless, the production of PHB in these mixotrophic cultures has been achieved concurrently with high-value phycocyanin. The continual addition of carbon in the fed batch approach has effectively increased the C:N ratio over time, leading to culture conditions that nudge the C assimilation biochemistry to the formation of this storage product. Clearly, additional work on understanding how to manipulate C:N ratios to favour bioplastic production and minimise the effects on phycocyanin synthesis are still required. In fact, simply looking at C:N ratios may not be enough. Hauf et al. [64] have suggested that an improved understanding of the redox status of the cell is a more useful way to determine if PHA accumulation is likely.

Allorganic compounds (acetate, oxalate and glycerol) investigated in this study can be found in one or more industrial waste streams. Being able to utilise the unrefined aqueous waste streams also enhances the carbon capture capabilities of the microalgal culture. The majority of previous studies have utilised either glucose or acetate as the alternative carbon source, including unrefined versions such as molasses (50% *w*/*w* sugars), for algal mixotrophic growth [65] and have achieved similar results to the current study. There are far fewer studies that have utilised alternate small organic acids, e.g., oxalate and formate [35], and none that appear to have specifically tested combinations of these organic acids and sugars/glycerol.

The fact that these non-axenic cultures showed growth that was significantly higher than phototrophic-only conditions, utilising such carbon sources as oxalate and the mixtures of glycerol and small organic acids, was encouraging. These results suggest that liquid/aqueous waste streams that contain a mixture of organic carbonaceous wastes can be effectively employed as a feed supplement in microalgal culture. This reduces the reliance of having to feed cultures on a single, refined carbon source, minimizing costs associated with pre-fractionation of waste streams before being directed into the microalgal cultivation systems.

Fed-batch techniques have been considered a useful strategy to enhance biomass growth and metabolite accumulation in many bioprocesses, as this strategy allows manipulation of substrate concentrations in order to maximise productivity [66]. This cultivation process also allows for a great deal of flexibility in regulating the supplementation of carbon sources to minimise toxicity and inhibition by medium constituents (including substrates) at elevated concentrations. Fed-batch culture modes are attractive as they favour a short turnaround time and high productivity for the overall bioprocess [67]. However, to sustain the hyper-production of the compounds of interest, the substrate quantity and time of feeding are crucial.

This is particularly relevant when attempting to produce multiple bioproducts from a single organism that are synthesised from different biochemical pathways. A more nuanced understanding of carbon fluxes and assimilation within individual species and strains would assist in determining which biochemical triggers can be manipulated in order to enhance production of these products.

The goal of sustainably mass cultivating microalgae such as *A. plantensis* under non-axenic conditions for multiple bioproducts, and being able to manipulate conditions to favour production of one or the other of these at any point in the cultivation, has yet to be realised. Our results, while promising, suggest that further, detailed knowledge of the interaction of biochemical pathways for particular products is needed to ascertain the best tools/conditions required to achieve production of multiple, high-value products at scale. This would result in better decision-making on what ‘macro/industrial-level’ culture conditions, e.g., nutrient status, supplemental carbon dosing, type of carbon used for supplementation, oxygen levels, etc., lead to enhanced production of high-value products, such as PHB and phycocyanin, from *A. platensis*.

## 4. Conclusions

Our aim was to investigate if a pulse-feeding approach to supplying supplemental organic carbon would result in a non-axenic *A. platensis* culture able to co-produce the two high-value products phycocyanin and PHB bioplastic. Furthermore, we wished to utilise organic carbon sources that could conceivably be present in agro-industrial wastewaters. The results showed that this approach led to increased biomass and bioplastic yield compared to photoautotrophic conditions alone, with minimal impact on phycocyanin productivity, for those cultures supplemented with small organic acids. However, the culture supplemented with glycerol-only failed and was subject to significant bacterial contamination, indicating that this substrate may not be useful for mixotrophic growth of non-axenic *A. platensis*. Our findings suggest that pulse-feeding a carbon supply can meet the nutritional requirements of the microalgal culture and avoids catabolism of accumulated PHB observed with a single bolus carbon source addition. However, it is not clear if the bioplastic is being produced by *A. platensis* or contaminant bacteria, or both.

There were indications that the use of the pulse-feeding approach leads to *A. platensis* metabolism switching from photoautotrophic to heterotrophic mode, and the combination of this stress plus enhanced bacterial growth can have detrimental impacts on culture health. This is particularly apparent in those cultures supplemented exclusively on a sugar (glycerol) where the culture crashed with significant loss of biomass and pigment production. Culture health and growth were more favourable when using small organic acids or combinations of small organic acids with glycerol as supplementary carbon sources.

A better understanding of the interactions of competing product biosynthetic pathways and finer control of the rate of carbon supplementation needs to be achieved to avoid significant bacterial contamination under axenic conditions and to achieve the goal of co-producing pigments and bioplastics.

## Figures and Tables

**Table 1:** Comparison of growth, biomass and phycocyanin (C-PC) productivity in mixotrophic culture of *A. platensis*. The same superscript letter down a column indicates results that are not statistically significantly different.

	Specific Growth Rate (d^−1^)	Maximum Specific Growth Rate (d^−1^) *	Biomass Productivity (mg/l/d)	Maximum Biomass Productivity (mg/l/d) *	Maximum C-PC Productivity (mg/l/d) **	PHB Productivity (mg/l/d)
Photoautotroph	0.09 ± 0.004 ^c^	0.12 ± 0.001 ^d^	111 ± 7 ^f^	130 ± 31 ^d^	36.7 ± 0.5 ^ab^	0 ^c^
Acetate	0.17 ± 0.008 ^a^	0.24 ± 0.009 ^a^	272 ± 8 ^a^	341 ± 9 ^a^	39.6 ± 0.1 ^a^	3.6 ± 1.1 ^b^
Oxalate	0.11 ± 0.002 ^bc^	0.14 ± 0.004 ^d^	152 ± 2 ^d^	177 ± 9 ^c^	36.3 ± 1.2 ^ab^	0 ^c^
Glycerol	0.09 ± 0.009 ^c^	0.23 ± 0.009 ^ab^	130 ± 7 ^e^	344 ± 16 ^a^	23.9 ± 1.2 ^d^	11.1 ± 1.7 ^a^
G + O	0.12 ± 0.003 ^b^	0.19 ± 0.002 ^c^	183 ± 1 ^c^	260 ± 11 ^b^	28.3 ± 0.1 ^c^	3.6 ± 0.7 ^b^
G + O + A	0.15 ± 0.006 ^a^	0.20 ± 0.009 ^bc^	227 ± 5 ^b^	259 ± 7 ^b^	34.6 ± 1.5 ^b^	3.9 ± 0.7 ^b^

* Maximum specific growth rate and maximum biomass productivity are calculated based on the amount of biomass at day 0 and 4 for all treatments. ** Maximum C-PC productivity is calculated based on the amount of C-PC at day 0 and 6 for photoautotroph, acetate and oxalate. However, for glycerol, G + O and G + O + A treatments, calculation was based on C-PC produced between day 0 and 4.

**Table 2:** PHB content and productivity of cyanobacteria under mixotrophic conditions.

Algal Species	Culture Medium	Culture Mode	Phycocyanin (% *w*/*w*)	Max PHB (% *w*/*w*)	PHB Productivity (mg/L/d)	Ref.
*Arthrospira platensis*	Zarrouk	Batch, photoheterotrophic, 0.5% *w*/*v* acetate, nitrogen deprivation	n.r	19.2 ± 0.5	14.5 ± 0.7	[53]
	Zarrouk	Batch, mixotrophic, 0.1% *w*/*v* butyrate, nitrogen deprivation	n.r	17.8 ± 0.7	12.5 ± 0.6	[53]
	Zarrouk	Batch, photoheterotrophic, 0.75% *w*/*v* glucose, nitrogen deprivation	n.r	15.4 ± 0.7	10.8 ± 0.8	[53]
	Zarrouk	Batch, photoheterotrophic, 0.1% *w*/*v* propionate, nitrogen deprivation	n.r	12.6 ± 0.5	8.9 ± 0.6	[53]
	Zarrouk	Batch, mixotrophic, 3.0–6.14 g/L pure glycerol	7.8 ± 3.3 to 4.2 ± 3.1	1.3 ± 0.6 to 1.1 ± 1.5	2.4–2.6	[54]
	Zarrouk	Batch, mixotrophic, 3.0–6.14 g/L crude glycerol	10.8 ± 4.3 to 2.7 ± 2.6	0.06 ± 0.001 to 0.5 ± 0.11	0.09–1.0	[54]
	Zarrouk	Batch, mixotrophic, acetate, formate, glycerol, oxalate, air ± CO_2_	11.0–14.5	0.1–3.0	0.2–7.8	[35]
	Zarrouk	Fed batch, mixotrophic, acetate	8.4 ± 0.1	1.5 ± 0.5	3.6 ± 1.1	This study
	Zarrouk	Fed batch, mixotrophic, oxalate	9.8 ± 0.1	0	0	This study
	Zarrouk	Fed batch, mixotrophic, glycerol	4.3 ± 0.2	7.8 ± 1.5	11.1 ± 1.7	This study
	Zarrouk	Fed batch, mixotrophic, glycerol + oxalate	6.3 + 0.3	1.5 ± 0.2	3.6 ± 0.7	This study
	Zarrouk	Fed batch, mixotrophic, glycerol + oxalate + acetate	6.9 + 0.2	1.9 ± 0.1	3.9 ± 0.7	This study
*Spirulina* sp. LEB18	Biopolymer extraction waste	Batch, mixotrophic, 25% *v*/*v* waste	n.r	10.6	4.7	[59]
*Nostoc muscorum*	ES	Batch, mixotrophic, 1% *w*/*v* glucose + 1% *w*/*v* acetate, phosphate deprivation	n.r	16.4 + 2.7	6.5	[60]
*Scytonema geitleri*	Chu 10	Batch, mixotrophic, 30 mM acetate		7.1	n.r	[61]
*Synechocystis* sp. PCC6803-OEphaAB	BG 11 + 30 g/L chloramphenicol	Batch, mixotrophic, 4 mM acetate, nitrogen deprivation	n.r	35	n.r	[62]
*Synechocystis* sp. PCC6803-ΔSphU	shrimp wastewater	Batch, mixotrophic	n.r	32.5 + 1.7	12.7	[63]

## Data Availability

The data presented in this study.

## Supplementary Materials

The following supporting information can be downloaded at: https://www.mdpi.com/article/10.3390/biotech12030049/s1, Figure S1: *A. platensis* cultures fed with different organic carbon sources at the last day of experiment; Figure S2: Bacterial contamination of different cultures using filtered or centrifuged biomass as inoculum at last day of each experiment; Table S1: The semi-quantitative data for bacterial loads in *A. platensis* cultures fed with different organic carbon sources.

## Abbreviations

PHA	polyhydroxyalkanoate
PHB	polyhydroxybutyrate
C-PC	phycocyanin
PUFA	polyunsaturated fatty acid
