# Supplementary material for: Co-Producing Phycocyanin and Bioplastic in Arthrospira platensis Using Carbon-Rich Wastewater
Source: BioTech (Basel). 2023 Jul 3;12(3):49. doi: 10.3390/biotech12030049 (PMC10366904; doi:10.3390/biotech12030049)
Supplement: Supplementary file 1 [file biotech-12-00049-s001.zip › biotech-2377348-supplementary.pdf]

Co-producing phycocyanin and bioplastic in *Arthrospira platensis* using carbon rich wastewater

Hajar Shayesteh, Damian W. Laird, Leonie J. Hughes, Mohammad A. Nematollahi, Amin Mirshamsi Kakhki and Navid R. Moheimani

Supplementary Information

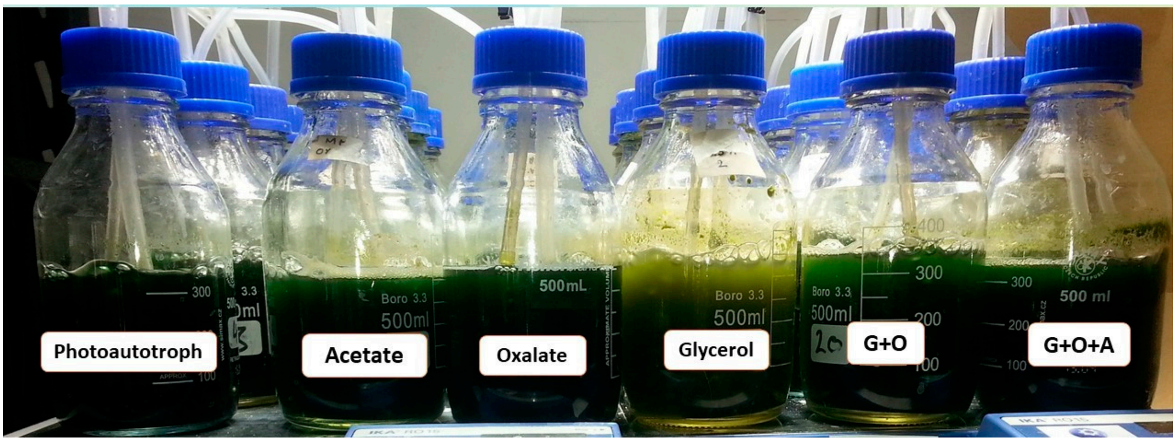

Figure S1. A. platensis cultures fed with different organic carbon sources at the last day of experiment.

Glycerol only culture shows visible discolouration.

Bacterial counts were conducted at the end of each experiment as the Arthrospira platensis source culture was non-axenic and most bacteria are capable of producing PHB. A 3M™ Petrifilm™ Aerobic Count Plate (http://www.3m.com.au) that contains a water-soluble gelling agent, nutrients and a red indicator dye, which makes colonies red for better contrast, was utilised. After preparing samples and making a 300x serial dilution, 0.5 mL of the dilute solution was plated on the Petrifilm plate and incubated at 37 ˚C for 48 h. Total colony forming unit (CFU) was calculated using equation:

CFU/mL= Number of colonies counted * Dilution factor/volume of diluted sample

Table S1. The semi-quantitative data for bacterial loads in *A. platensis* cultures fed with different organic carbon sources.

Treatments	Bacteria count (x10^3 cfu/ml)	Semi-quantitative data
Photoautotroph	1 ± 0.35^{f}	+
Acetate	97 ± 3.9^{c}	++++
Oxalate	34 ± 3.9^{e}	++
Glycerol	235 ± 5.0^{a}	++++++
G + O	116.4 ± 6.9^{b}	+++++
G + O + A	51.6 ± 3.3^{d}	+++

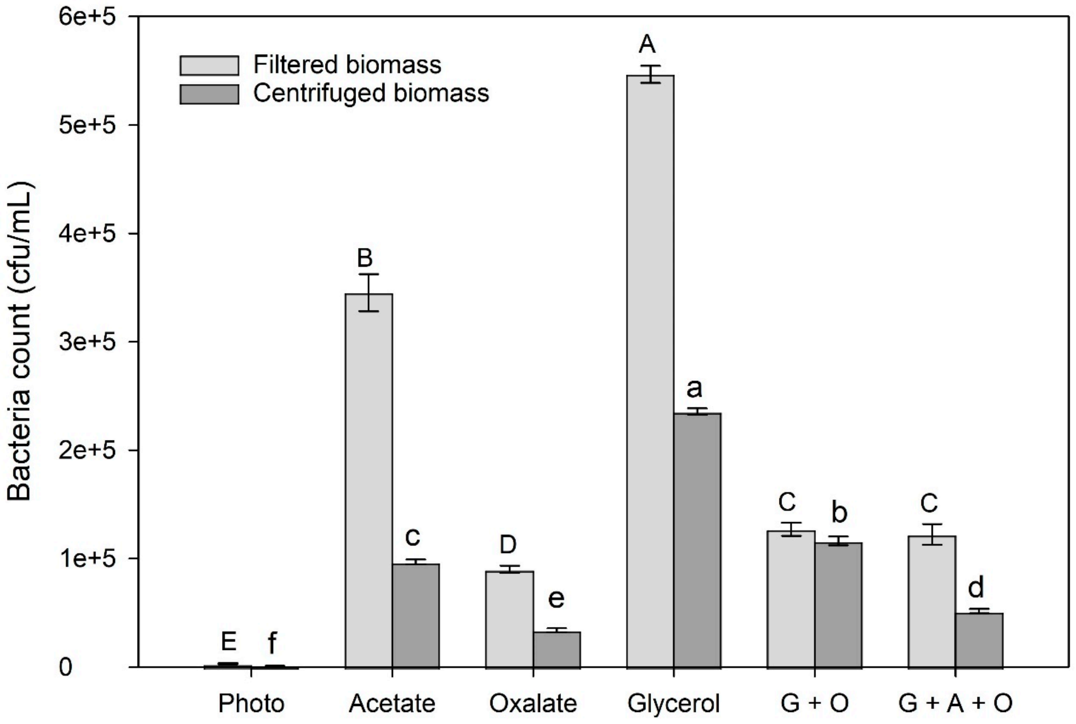

Figure S2. Bacterial contamination of different cultures using filtered or centrifuged biomass as inoculum at last day of each experiment. A difference in the uppercase letter within light grey bars (i.e., filtered biomass inoculum) indicates a significant difference between carbon source. Different lowercase letters within dark grey bars (centrifuged biomass inoculum) indicates a significant difference between carbon source.
